# Effects of Atorvastatin on Nitrate Tolerance in Diabetic Rats

**Published:** 2010

**Authors:** Mohsen Imenshahidi, Gholamreza Karimi, Ehsan Kazemzadeh

**Affiliations:** *School of Pharmacy, Mashhad University of Medical Sciences, Mashhad, Iran.*

**Keywords:** Diabetes, Streptozotocin, Nitroglycerin, Atorvastatin, Nitrate tolerance

## Abstract

Statins have been reported to show preventive effect on nitrate tolerance in normal rats, but there are no reports on their effect in diabetic animals. In this study, diabetes was induced in male wistar rats by a single intraperitoneal injection of streptozotocin (45 mg/kg). Five groups of diabetic and five groups of normal rats were treated; groups 1 (of normal and diabetic rats) received atorvastatin (10 mg/kg/d p.o. for 8 weeks) and groups 2 received atorvastatin (10 mg/kg/d p.o. for last 3 days). Groups 3 and groups 4 were similar to groups 1 and 2 respectively, except that they received nitroglycerin (50 mg/kg/d, b.i.d. for last 3 days of the study). Groups 5 received neither atorvastatin nor nitroglycerin. After 8 weeks, relaxations to nitroglycerin (0.01 to 10 nM) and nitroprusside (0.01 to 10 nM) were determined on phenylephrine-preconstricted aortic rings. The relaxation response to nitroglycerin in diabetic and normal aorta were not significantly different. The results showed that 8 weeks treatment with atorvastatin prevents nitrate tolerance in diabetic and normal rats, but in nitrate tolerant animals, 3 days treatment with atorvastatin was not effective on protection against nitrate tolerance.

## Introduction

Apart from cholesterol-lowering effects, HMG CoA reductase inhibitors exert many pleiotropic effects on the vascular wall. They have beneficial effects on endothelial function and blood flow, decrease low density lipoprotein (LDL) oxidation, enhance the stability of atherosclerotic plaques, inhibit vascular smooth muscle cell proliferation and platelet aggregation, adverse left ventricular remodeling, decrease serotonin-induced contractions in coronary arteries and also reduce vascular inflammation (1-3).

In animal studies, statins protect nitrate tolerance by counteracting nitroglycerin-induced increase in O- production. Both eNOS pathway and NAD(P)H oxidases seems to be involved in this protective mechanism (4).

Chronic diabetes affects endothelial function via eNOS pathway and NAD(P)H oxidases (5, 6). In this study we investigated the effect of statins on nitrate tolerance in diabetic animals. 

## Experimental

**Materials**

Phenylephrine hydrochloride, nitroglycerin (NTG) and sodium nitroprusside (SNP) were obtained from Sigma. Sodium chloride, potassium chloride, magnesium sulfate, sodium hydrogen carbonate, potassium hydrogen orthophophate, D-glucose, sodium chloride and calcium chloride were purchased from Merk laboratories. Sodium thiopental was obtained from Biochemie GmbH (Vienna, Austria). Streptozotocin (STZ) was obtained from Pharmacia & Upjohn (USA). 

*Animals*

In this study, we used male wistar rats (Razi Institutes, Mashhad, Iran), weighing 250-300 g. All animal procedures were approved by the ethical committee of Mashhad University of Medical Sciences. 

*Study design*

Diabetes, induced in male wistar rats (Razi Institutes, Mashhad, Iran) weighing 250-300 g, by a single intraperitoneal injection of streptozotocin (45 mg/kg) dissolved in sterile 0.9% NaCl solution, was verified 24 h later by estimating hyperglycaemia (7). Non-diabetic control animals were injected with an equivalent volume of the vehicle. Both groups were maintained under the same conditions, supplied with food and water ad libitum until death. In some groups, animals received atorvastatin by drinking water. Manifestation of diabetes was verified one week later by determination of blood glucose by glucometer and test strips (Lifescan, Inc, USA). Rats were considered as diabetic when blood glucose levels were more than 270 mg/dL (8).

In our experiment, we used four groups of normal animals and four groups of diabetic animals. We designed protocols to evaluate the effect of 8 weeks or 3 days atorvastatin on nitrate tolerance in diabetics and normal tolerant and non-tolerant animals. Tolerant animals have reduced response to nitroglycerine due to three days of nitroglycerine administration, while non-tolerant animals have normal response to nitroglycerine. The protocols used for each group of animals were summarized in Table 1.

**Table 1:** Protocols of the treatment* of animal throughout eight weeks

Group	Single injection of STZ (45 mg/kg) in the first day	Atorvastatin (in drinking water) for 8 weeks	Atorvastatin (in drinking water) in the last 3 days	NTG (50 mg/kg/d, s.c. injections b.i.d.) in the last 3 days
Normal 1	-	-	-	-
Diabetic 1	+	-	-	-
Normal 2	-	+	+	-
Diabetic 2	+	+	+	-
Normal 3	-	-	-	+
Diabetic 3	+	-	-	+
Normal 4	-	+	+	+
Diabetic 4	+	+	+	+
Normal 5	-	-	+	+
Diabetic 5	+	-	+	+

+ Administered

- Not administered

*Studies of vasomotor responses*

At the end of 8 weeks treatment periods, rats were anesthetized with interaperitoneal injection of sodium thiopental (80 mg/kg) and their thoracic aorta was removed, cleaned of adhering fat and cut into rings of 3-4 mm long. All rings were mounted under 2 g resting tension on stainless steel hooks in 20 mL organ baths. These organ chambers were filled with Krebs- Henseleit solution (KHS), with a composition (in mM) of: NaCl 118, KCl 4.7, MgSO_4_ 2 H_2_O 1.2 KH_2_PO_4_, 2 H_2_O 1.2, NaHCO_3_ 25, CaCl_2_ 2.5 and glucose 11.1., aerated with a mixture of 95% O_2_ /5% CO_2_ and kept at 37°C. Tension was measured isometrically by a force transducer (Grass FTO3C) and recorded continuously by a transducer amplifier (Janssen Scientific Instruments) and a pen recorder. 

Aortic rings were pre-contracted with 1 μM phenylephrine and concentration-response curve for nitroglycerin NTG (0.01 to 10 nM) and SNP (0.01 to 10 nM) were obtained by cumulative addition of these drugs to the bath solution. These experiments were performed in all 8 groups of normal and diabetic groups

*Statistical analysis of data*

Results were expressed throughout as Means ± S.E.M. and were analyzed by one-way analysis of variance (ANOVA) followed by a Tukey-Kramer multiple comparison test. A P value of <0.05 was considered to be significant.

## Results

*Blood glucose levels in normal and diabetic rats*

Streptozotocin treatment significantly increased blood glucose concentration (Table 2). But atorvastatin had no significant effects on blood glucose in normal or diabetic animals (P > 0.05). 

**Table 2:** Blood glucose concentrations (mg/dL) in control and diabetic rats, 2 and 8 weeks after treatment with either saline or streptozotocin (n = 15–20).

Group	2 Weeks	8 Weeks
Control	161 ± 12	158 ± 15
Diabetic	315 ± 10^a^	295 ± 14^a^

*Induction of nitrate tolerance in normal and diabetic groups*

**Figure 1:**
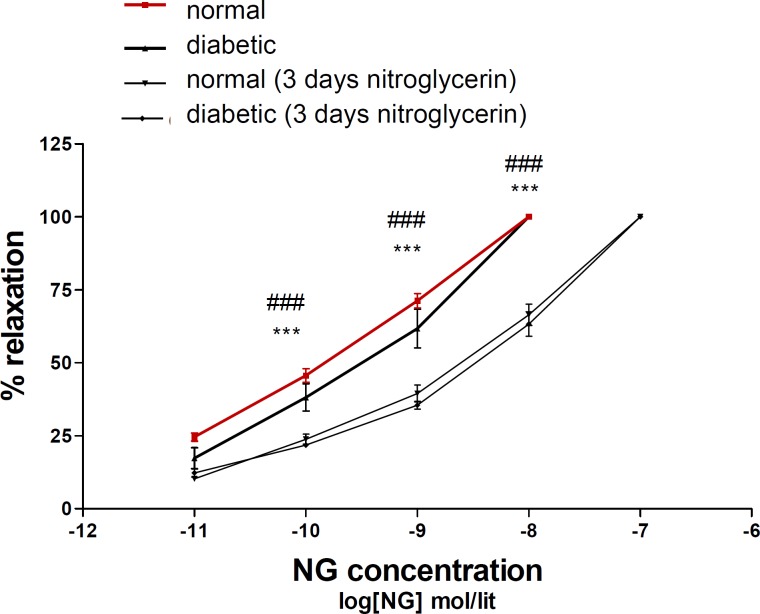
Concentration–response curves to nitroglycerin (NTG) in the phenylephrine (1 μM) pre-contracted aortic rings isolated from normal rats (■), diabetic rats (▲), normal rats treated with NTG for 3 days (▼) and diabetic rats treated with NTG for 3 days (♦). Data are mean ± SEM. A statistically significant difference was observed between normal (3 days NTG) rats comparing with normal rats not exposed to NTG. ***P < 0.001. There is also significant difference between diabetic (3 days NTG) rats comparing with diabetic rats not exposed to NTG. ### P < 0.001

Relaxation response to nitroglycerin in diabetic animals was similar to normal animals. Three days administration of nitroglycerin induced nitrate tolerance in both of normal and diabetic groups (Figure 1).

*Effect of atorvastatin treatment on nitrate tolerance in normal animals*

**Figure 2:**
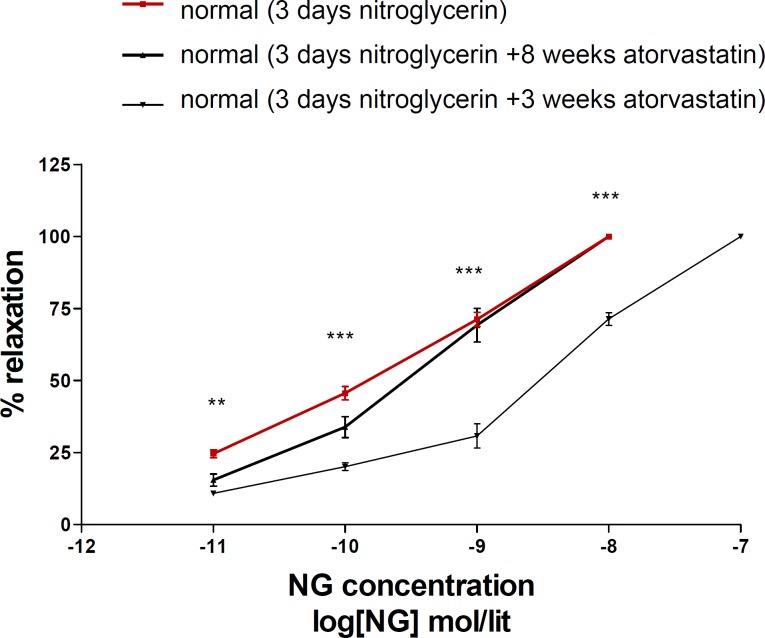
Concentration–response curves to nitroglycerin (NTG) in the phenylephrine (1μM) pre-contracted aortic rings isolated from normal rats exposed to NTG for 3 days (■), normal rats treated with atorvastatin (10 mg/kg p.o.) for 8 weeks and exposed to NTG for 3 days (▲) and normal rats treated with atorvastatin (10 mg/kg p.o.) for 3 days and exposed to NTG for 3 days (▼). Data are mean ± SEM. A statistically significant difference was observed between normal (3 days NTG) rats comparing with normal rats (3 days NTG) treated with atorvastatin (10 mg/kg/d p.o.) for 3 days. ***P<0.001; **P < 0.01. There is not significant difference between normal (3 days NTG) rats comparing with normal rats (3 days NTG) treated with atorvastatin (10 mg/kg/d p.o.) for 8 weeks (P > 0.05).

Eight weeks administration of atorvastatin prevented nitrate tolerance induction in normal animals, but 3 days atorvastatin was not effective on nitrate tolerance (Figure 2).

*Effect of atorvastatin treatment on nitrate tolerance in diabetic animals*

**Figure 3:**
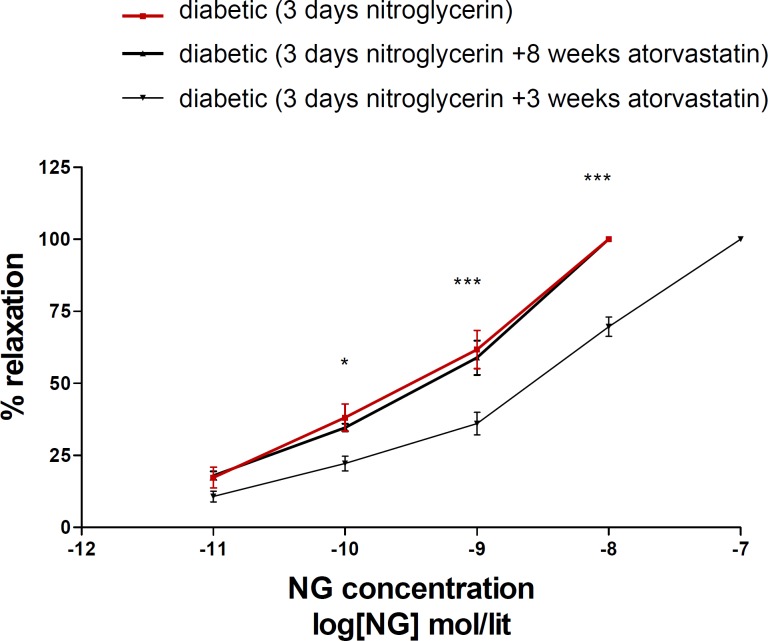
Concentration-response curves to nitroglycerin (NTG) in the phenylephrine (1 μM) pre-contracted aortic rings isolated from diabeticl rats exposed to NTG for 3 days (■), diabetic rats treated with atorvastatin (10 mg/kg/d p.o.) for 8 weeks and exposed to NTG for 3 days (▲) and diabetic rats treated with atorvastatin (10 mg/kg p.o.) for 3 days and exposed to NTG for 3 days (▼). Data are mean ± SEM. A statistically significant difference was observed between diabetic (3 days NTG) rats comparing with diabetic rats (3 days NTG) treated with atorvastatin (10 mg/kg/d p.o.) for 3 days ***P < 0.001; **P < 0.01; *P < 0.05. There is not significant difference between diabetic (3 days NTG) rats comparing with diabetic rats (3 days NTG) treated with atorvastatin (10 mg/kg p.o.) for 8 weeks (P > 0.05).

Eight weeks administration of atorvastatin prevented nitrate tolerance induction in diabetic animals; but 3 days atorvastatin was not effective on (Figure 3). 

*Responsiveness to nitroprusside*

Administration of nitroglycerin (3 days) and administration of atorvastatin (3 days or 8 weeks) had effects on responsiveness of aorta to nitroprusside (data not shown). 

## Discussion

The results showed that atorvastatin protects normal and diabetic rats against nitrate tolerance on eight- week treatment protocol, but short-term (3 days) administration of atorvastatin is not as effective. To the best of our knowledge, this is the first report on the evaluation of duratuion of atorvastatin administration on prevention of nitrate tolerance in both normal and diabetic rats. Similar results have been previously obtained in normal rats (4), but to the best of our knowledge, this is the first report on the same effect in diabetic animals. This particularly important as diabetes is one of the pathologic conditions that affect endothelial response to the vasoldilator agents (9). 

Although there are two published studies that showed a primary nitrate tolerance in diabetic patients (10, 11), however, based on our data, it seems that 8 weeks diabetes did not reduce the relaxatory response to nitroglycerin by itself. Three days administration of nitroglycerin induces tolerance in both normal and diabetic animals. This discrepancy may be related to species differences, the duration of diabetes or difference in type of study (in vivo versus in vitro).

No change in response of aorta to nitroglycerin was observed after 8 weeks administration of atorvastatin. Thus, it is assumed that atorvastatin does not show direct effects on relaxatory response of aorta, but prevents nitrate tolerance induction. Administration of atorvastatin had also no effects on the blood sugar of diabetic nimals. Therefore, the protective effect of atorvastatin is not related to changes in diabetic conditions. 

The response of nitrate tolerant aorta to nitroprusside (as a NO donor) was not significantly different in both group of nitrate tolerant and atorvastatin treated animals. Thus, it can be deduced that nitrate tolerance is not related to decrease in responsiveness of aorta to NO and the preventive effect of atorvastatin is not caused by enhancement of NO responsiveness of aorta. 

These findings are interesting as previous studies showed that neither diabetes (7) nor nitroglycerin (12) may change the sensitivity of smooth muscle to NO. Other factor that can change the responsiveness of aorta to nitroglycerin, is improvement of NO bioavailability, which can be achieved either by increasing of NO production via NOS stimulation or by reducing NO inactivation by oxidative radicals. The first mechanism (i.e. stimulation of NOS synthase), does not seem to be important in these circumstances because 8 weeks administration of atorvastatin did not changed the responsiveness of non-nitrate tolerated aorta to the nitroglycerin. But, regarding the second mechanism (i.e. reducing oxidative stress), it is reprted that nitrate tolerance increases super oxide production. Statins impede the production of superoxide anion via eNOS pathway (4). Meanwhile, statins inhibit Rac GTPase activity via inhibition of geranyl geranylation, which decreases NAD(P)H-oxidase activation and superoxide production (14-16). Other studies also showed direct antioxidant activity of statins that can contributes in protective effect of atorvastatin on NO degradation by super oxide anion (17, 18). Among these mechanisms for reducing oxidative stress, it seems that direct antioxidant activity of statins is not important in these conditions and inhibition of Rac GTPase activity via geranyl geranylation inhibition seems to have more important role; because inhibition of geranyl geranylation is a slow mechanism needs time. This is consistent with our results that showed long term (8 weeks) but not short term (3 days) administration of atorvastatin has protective effect against nitrate tolerance. 

In conclusion, the present study shows that diabetes can not induce nitrate tolerance by itself and 8 weeks administration of atorvastatin can prevent nitrate tolerance in diabetic as well as normal rats.
